# Negative Serum Ascites Albumin Gradient (SAAG) in the Setting of Cholangiocarcinoma: A Case Report

**DOI:** 10.7759/cureus.37528

**Published:** 2023-04-13

**Authors:** Emily Shilakis, Raghav Bassi, Pranav Prakash, Eason Balakrishnan, Almari Ginory, Matthew Calestino

**Affiliations:** 1 Psychiatry, University of Central Florida College of Medicine, Graduate Medical Education/North Florida Regional Medical Center, Gainesville, USA; 2 Internal Medicine, University of Central Florida College of Medicine, Graduate Medical Education/North Florida Regional Medical Center, Gainesville, USA, Gainesville, USA; 3 Internal Medicine, University of Central Florida College of Medicine, Graduate Medical Education/North Florida Regional Medical Center, Gainesville, USA

**Keywords:** cholangiocarcinoma, cholangiocarcinoma diagnosis, malignant ascites, gi malignancy, negative saag

## Abstract

Ascites is the accumulation of fluid in the peritoneal cavity which leads to abdominal distention. Malignant ascites may occur in several tumor types including liver, pancreas, colon, breast, and ovary. Serum ascites albumin gradient (SAAG) is the difference between albumin in the serum and ascitic fluid. A SAAG greater or equal to 1.1 g/dL is characteristic of portal hypertension. A SAAG less than 1.1 g/dL can be seen in hypoalbuminemia, malignancy, or an infectious process. We report a rare case of malignant ascites in a 61-year-old female patient who presented with a chief complaint of abdominal pain with distention that was preceded by a 25-pound weight loss over the last three months. The patient underwent a paracentesis after a computed tomography (CT scan) revealed a heterogenous liver mass with associated ascites. Ascitic fluid analysis revealed a SAAG of -0.4 g/dL. CT-guided core needle biopsy of the hepatic mass revealed a poorly differentiated carcinoma with immunostaining suggestive of an underlying cholangiocarcinoma. Cholangiocarcinoma is an extremely uncommon etiology of acute new-onset ascites and has not been shown to produce high protein ascites with a negative SAAG. It is therefore important for clinicians to get ascitic fluid analysis in order to calculate a SAAG to help develop differential diagnosis for the cause of ascitic fluid buildup.

## Introduction

Approximately 80% of patients with ascites in North America and Western Europe result from underlying cirrhosis [1]. It has been reported that within 10 years, 50% of patients with decompensated cirrhosis will develop ascites and die within two years [1,2]. Mortality increases from complications such as spontaneous bacterial peritonitis (SBP) and from hepatorenal syndrome [1]. Other less common causes of ascites include cancer which accounts for about 10% of cases, heart failure makes up 3% of cases, tuberculosis 2% of cases, and pancreatic disease a mere 1% of cases [1,2]. Evaluation of patients with new onset ascites generally begins with a comprehensive history and physical examination followed by a diagnostic and often therapeutic paracentesis. Evaluation of ascitic fluid includes total protein, white blood cell count with differentials, and albumin for the calculation of a serum-ascites albumin concentration (SAAG) [1,3]. Other tests can also be ordered based on clinical suspicion such as cytology if an underlying malignancy is suspected or amylase for suspected pancreatic ascites. SAAG is calculated by taking the serum albumin and subtracting it from the albumin level in the ascitic fluid. A SAAG greater than 1.1 g/dL is highly sensitive and specific to ascites from increased pressures within the portal venous system causing portal hypertension (sensitivity of 97% and specificity of 90.2%) [3]. In addition, cell count with differentials is essential to diagnose spontaneous bacterial peritonitis (SBP) which is the most common infection of the ascitic fluid and can be fatal if not treated. SBP occurs primarily due to Gram-negative bacterial translocation, a common malady seen in patients with cirrhosis [3,4]. Cell counts can also be helpful in diagnosing malignancy-related ascites, as patients typically present with a fluid white blood count (WBC) of more than 500 leukocytes/mm^3^ and a SAAG of less than 1.1 g/dL with a total protein of 2.5 g/dL or greater due to the production of proteinous fluid by the tumor cells [3]. Peritoneal carcinomatosis accounts for two-thirds of patients with malignancy-related ascites with adenocarcinomas being the most commonly associated malignancy [1]. Cholangiocarcinoma, on the other hand, is extremely rare and represents less than 3% of all gastrointestinal tumors with ascites and jaundice being a late sequela of the disease [4]. Herein, we present an extremely rare case of a patient whose ascitic fluid analysis was significant for a negative SAAG and histological testing of her liver mass confirmed an underlying cholangiocarcinoma.

## Case presentation

A 61-year-old Caucasian female with a past medical history of hypertension, heart failure with reduced ejection fraction, and diabetes mellitus type 2 presented to the emergency department (ED) for abdominal pain, constipation, and distension.

The patient initially presented to the ED a month prior to her recent visit with a three-week history of sharp non-radiating left flank pain. On evaluation, she was found to have a urinalysis suggestive of a urinary tract infection (UTI). Additionally, abdominal imaging revealed an indeterminate mass in the right lobe of her liver measuring 3.9 x 3.1 cm. She was treated with intravenous antibiotics for her UTI for a week and advised to follow up with her primary care doctor and obtain a gastrointestinal evaluation to evaluate her liver mass further.

**Figure 1:**
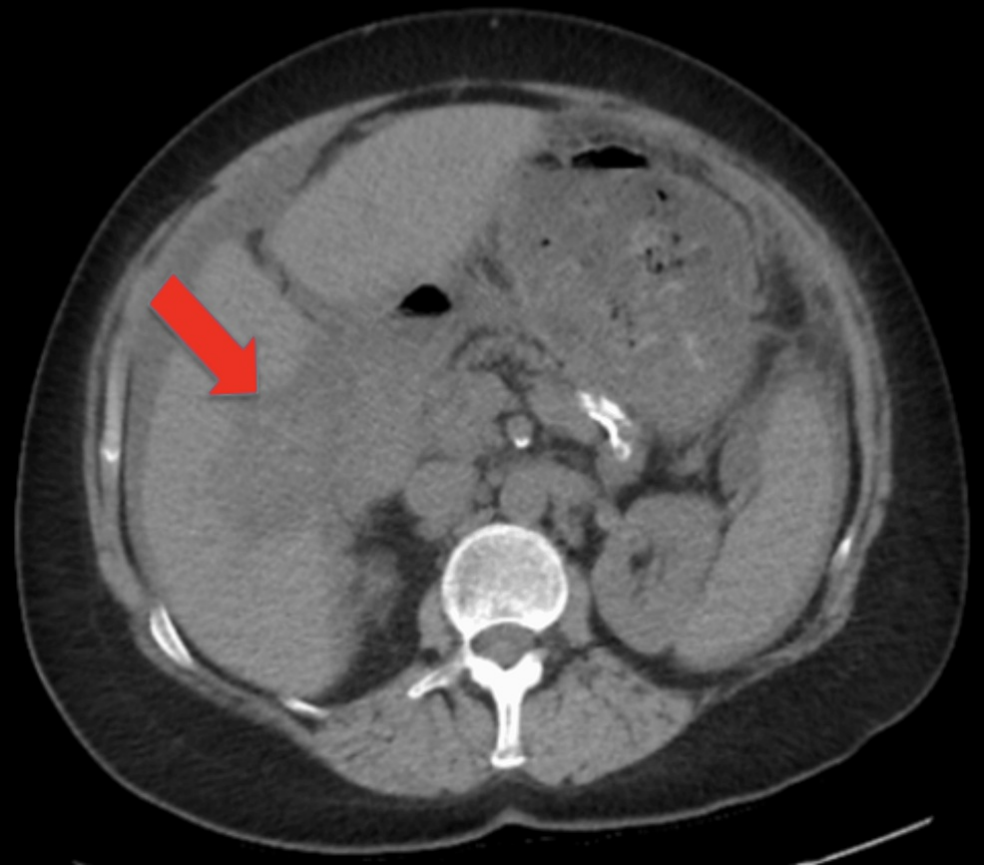
Initial CT scan of the abdomen and pelvis. The image demonstrates an ill-defined hepatic mass measuring approximately 5.9 x 3.9 cm with increased soft tissue attenuation noted along the posteromedial aspect of the surface of the right lobe of the liver.

She now presented to the ED with complaints of worsening abdominal pain, characterized as 8/10 in intensity, associated with significant abdominal distention. She also reported additional symptoms, such as constipation, urinary frequency, urinary urgency, pelvic pain, and a weight gain of 25 pounds in three months. On physical examination, the patient was noted to have active bowel sounds, soft but tender distended abdomen, and negative jugular vein distention or lower extremity edema. Computed tomography (CT) abdomen and pelvis at this visit demonstrated a liver mass measuring 5.9 x 3.9 cm, representing a 2 cm increase in size within the last four weeks. She also had increased attenuation of the omentum within the mid and lower abdomen and interval development of moderate ascites (Figure 1). During her current admission, routine labs were also significant for elevated creatinine of 1.54 mg/dL compared to her baseline of 1.1 mg/dL, elevated alkaline phosphatase of 290 units/L, and a decreased albumin level of 2.1 g/dL.

The patient had no previous history of liver disorders and viral hepatitis serology was negative. She was an active tobacco user with an 84-year pack history of smoking and denied alcohol use. She was admitted for further evaluation of her heterogeneous mass in the right lobe of the liver and an acute kidney injury (AKI). The rapid increase in the size of the mass was concerning for a neoplasm with hepatic abscess being in the differential diagnosis.

An ultrasound-guided therapeutic and diagnostic paracentesis was done on her second day of admission where 1700cc of amber-colored fluid was removed. Fluid studies revealed a WBC of 30,500 leukocytes/mm^3^ with 82% polymorphonuclear cells (PMN). Fluid albumin was 2.3 g/dL and lactate dehydrogenase (LDH) was 1512 units/L (Table 1). Her serum albumin measured during the same day was 1.9 g/dL corresponding to a SAAG of -0.4 g/dL (Table 2). Cytology and cultures were also sent to the lab for further evaluation.

**Table 1:** Ascites fluid analysis following diagnostic and therapeutic paracentesis suggestive of an underlying exudative effusion.

Lab test	Ascitic fluid analysis
Fluid color	Red
Fluid appearance	Cloud
Fluid WBC (cells/mm^3^)	30,500
Fluid red blood cells (cells/mm^3^)	68,000
Fluid segmented neutrophils (%)	82
Fluid lymphocytes (%)	14
Fluid eosinophils (%)	1
Fluid glucose (mg/dL)	170
Fluid protein (g/dL)	5
Fluid albumin (g/dL)	2.3
Fluid lactate dehydrogenase (units/L)	1512
Fluid amylase (units/L)	13
Fluid triglycerides (mg/dL)	49

**Table 2:** Calculated negative SAAG ruling out underlying portal hypertension. SAAG: serum-ascites albumin concentration

Lab test	Value
Serum albumin (g/dL)	1.9
Ascitic albumin (g/dL)	2.3
Calculated SAAG score (g/dL)	-0.4

**Figure 2:**
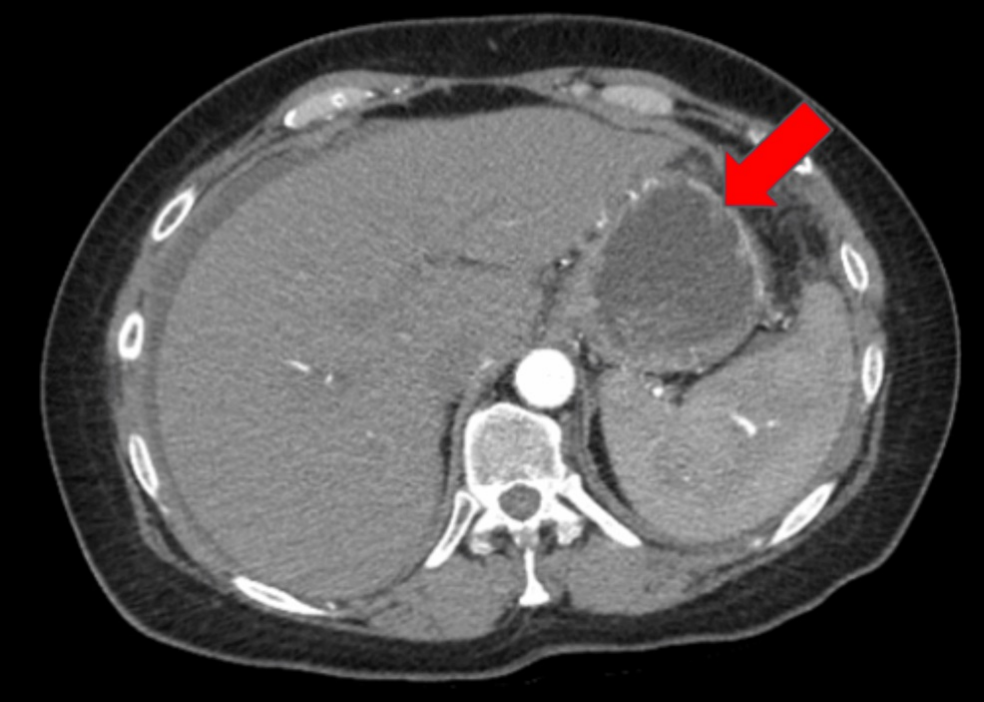
A triphasic CT scan of the liver in the venous phase, revealing increased contrast uptake of the hepatic mass suggestive of an underlying malignancy.

Due to the possibility of SBP as seen by significantly elevated WBC and neutrophil count, the patient was started on 1 g of intravenous (IV) ceftriaxone for seven days (Table 1). She also received IV fluids for her AKI and morphine for pain control. On day three of her hospitalization, tumor markers were obtained which revealed an elevated cancer antigen (CA) 125 to 360 units/mL and CA19-9 to 278 units/mL. Triphasic CT of the liver was obtained for further evaluation of her hepatic mass and it showed a heterogeneous contrast uptake which was suggestive of a neoplasm rather than an abscess (Figure 2). She then underwent a CT-guided core needle liver biopsy for a definitive diagnosis with immunostaining for different tumor markers.

**Figure 3:**
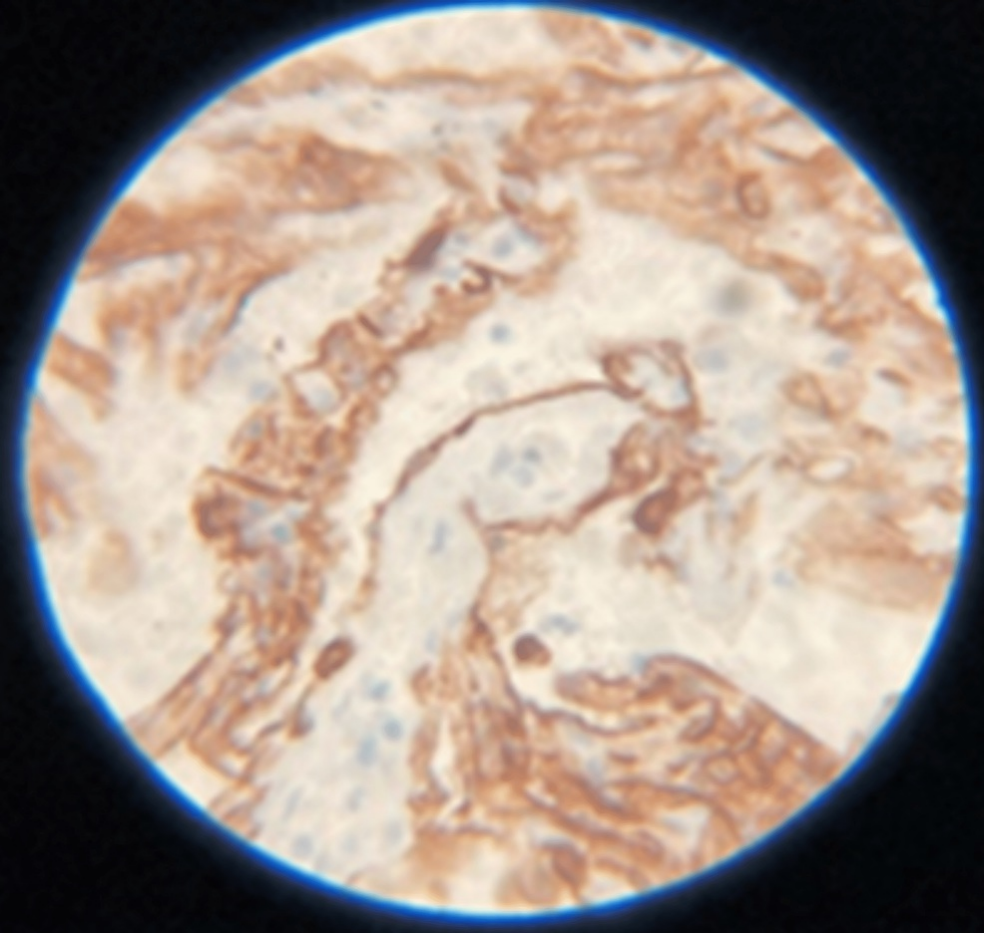
Malignant cells found in ascitic fluid aspirate demonstrating strongly positive CK 7 staining. CK 7 positive cells are found in a wide variety of carcinomas including lung, breast, thyroid, pancreas, and organs of the female genital tract.

The patient continued to complain about abdominal/discomfort and significant constipation. An abdomen x-ray was completed and it revealed a small bowel obstruction with no obvious transition point. She then received laxatives and suppositories to stimulate a bowel movement and to rest her bowels with an improvement in her symptoms. On day five of hospitalization, the cytological results of the ascitic fluid studies were positive for malignant cells which stained positive for cytokeratins (CK)7 immunostain (Figure 3). 

**Figure 4:**
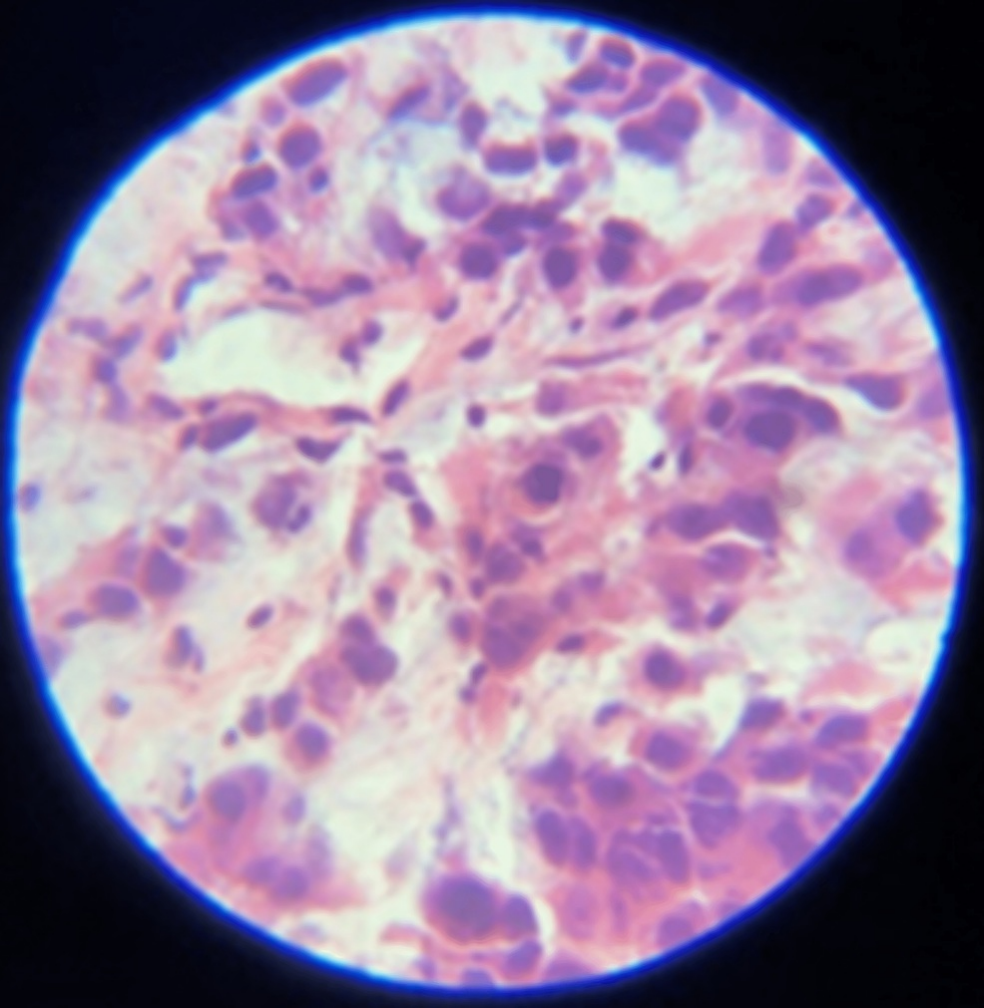
Cells obtained from a core biopsy of hepatic mass reveal hyperchromatic nucleoli of malignant cells. Due to the cells staining positive for CAM 5.2, CDX2, and CK20, a cancer of pancreaticobiliary or upper gastrointestinal tract origin was favored.

The liver biopsy identified tumor cells that were positive for CAM 5, 2, CK 7, and CDX2 on immunohistochemical staining and negative for HepPar, Glypican 3, GATA-3, PAX-8, and TTF-1 (Figure 4). Based on these findings, it was determined that the neoplasm was a poorly differentiated carcinoma of pancreaticobiliary or upper GI tract origin suggestive of an underlying cholangiocarcinoma. Ascitic fluid culture was also negative so antibiotics were discontinued as it was determined that the elevated WBC count in the ascitic fluid was more likely due to peritoneal carcinomatosis than an infection.

A multidisciplinary team comprising internal medicine, general surgery, oncology, radiology, and palliative care was involved in the management of the patient and after discussions with the patient, it was determined that the patient would need to pursue further imaging with a positron emission tomography scan to stratify the extent of her disease and have a chemotherapy port placed to initiate chemotherapy in the outpatient setting. The patient was discharged home in stable condition with family support.

Unfortunately, the patient was readmitted 10 days later with significant large-volume ascites and hyperkalemia. She required urgent hemodialysis and underwent large-volume paracentesis with multiple infusions of albumin. She had re-accumulation of her ascitic fluid post-procedure but was unable to undergo a repeat paracentesis due to persistent hypotension. Due to her aggressive disease course, the patient declined further aggressive treatment and elected to be transitioned to hospice care.

## Discussion

The analysis of ascitic fluid is vital to help establish a definitive diagnosis. The SAAG gives us insight into the pathophysiological basis of the underlying disease process by identifying the presence of portal hypertension with a sensitivity of up to 97% [5]. Our patient in this study had a SAAG of less than 1.1 g/dL which is indicative of an absence of portal hypertension. The next step in the evaluation of ascitic fluid should be the careful assessment of WBC and cell differentials, which in our patient were notable for a WBC count of 30,500 cells/mm^3 ^and PMN of 25,010 cells/mm^3^. These findings in isolation would raise suspicion about SBP, however, SBP is however exceedingly rare in patients without underlying cirrhosis and portal hypertension. It is essential to remember that 8% of malignancy-related ascites can have elevated ascitic WBC counts and PMN>250 cells/mm^3^, mimicking SBP [5]. Clinically, this can often lead to misdiagnosis and administration of antibiotics in patients whose disease is inherently the result of malignancy.

In patients with ascites studies revealing a low SAAG, it is imperative to pursue further diagnostic workup for peritoneal carcinomatosis to avoid misdiagnosis of SBP. A negative SAAG can either be the result of a low serum albumin state or a high ascitic albumin disease state as seen in certain malignancies. Therefore, other diseases that should remain on the differential diagnosis include heart failure, nephrotic syndrome, and pancreatic ascites based on individual patient risk factors, history, physical examination, and laboratory/radiographic findings. Our patient’s initial CT of the abdomen and pelvis scans revealed a hepatic mass in close proximity to the gallbladder which was suggestive of a neoplastic process or an abscess, though the presence of omental caking raised our suspicion for underlying malignancy. The clinical and radiographic features of this case are highly characteristic of peritoneal carcinomatosis. Given the high suspicion of a malignancy, a cytological evaluation of the ascitic fluid was done which confirmed the evidence of malignant cells. The low SAAG, in this case, was likely due to decreased resorption of ascitic fluid from the peritoneal cavity due to obstruction of subperitoneal lymphatic channels by tumor cells [6].

Adenocarcinomas account for 82% of malignancy-related ascites, among which adenocarcinomas of the breast, colon, stomach, pancreas, and lung comprise nearly 80% of cases [7]. Furthermore, what makes our case even more intriguing is the fact that immunohistochemical stains from the liver biopsy coupled with an elevated serum CA19-9 were highly suggestive that the primary tumor in our patient was a cholangiocarcinoma, which is an infrequent cause of ascites. The discovery of the primary site of the tumor is important as it influences therapeutic decisions and has significant prognostic implications. Omental caking is a radiographic feature often seen with intraperitoneal cancers and can present as a smudged or permeated appearance of the omental fat and as enhancing soft tissue nodules with disease progression as seen in Figure 2 [5,6]. It is often associated with metastatic disease from ovarian and gastric cancers, however, any tumor can cause it either through direct extension into the peritoneal ligaments, through intraperitoneal seeding, or through hematogenous/lymphatic spread [5]. Hepatobiliary malignancies, such as cholangiocarcinomas, are extremely unusual causes of omental caking, however, they can occur through direct invasion of the tumor along the hepatoduodenal ligament and lesser omentum as seen in our case above [5].

Cholangiocarcinomas that arise in the peripheral bile ducts within the liver parenchyma often reach a large size before becoming clinically evident. Patients present with hepatomegaly, back and abdominal pain, upper abdominal mass, and weight loss as seen in this case [6]. Ascites and painless jaundice are late sequelae of disease progression of intrahepatic cholangiocarcinomas due to tumor invasion of the hepatic ducts and intraperitoneal spread. In contrast to hepatocellular carcinoma, serum alpha-fetoprotein is only abnormal in less than 5% of patients, with 80% of patients having an elevated CA19-9 as seen in our patients in this study [6]. Hypercalcemia can also be seen due to parathyroid hormone-related protein by cholangiocarcinoma [6].

Diagnosing cholangiocarcinoma can be difficult as evaluation typically starts with ultrasonography (US) for suspected biliary obstruction and liver disease [6,7]. Intrahepatic cholangiocarcinoma may be identified as a mass lesion on ultrasound with an 87-96% sensitivity of detecting ductal masses or mural thickening. As it pertains to hilar and extrahepatic cholangiocarcinomas, visualization of the tumor is highly dependent on the skill of the sonographer. Another imaging modality that can be utilized in the detection of cholangiocarcinoma is a multidetector CT scan which can reveal hyperenhancement during the portal venous phase of the stenosed duct as seen in Figure 2. However, this modality has a specificity of only 19%. Furthermore, magnetic resonance imaging (MRI) with magnetic resonance cholangiopancreatography (MRCP) is usually considered the modality of choice with sensitivities and specificities over 90%. MRI is able to capture a high-resolution, multiplanar image and is further able to delineate between parenchyma, biliary and vascular extension. In addition, MRCP is able to differentiate between benign and malignant bile duct strictures giving further important diagnostic information [7]. When there is a high clinical suspicion of intrahepatic cholangiocarcinoma, an upper and lower endoscopy is recommended to rule out primary gastrointestinal tumors as these cancers can mimic colonic adenocarcinoma. Importantly, immunostaining for CK 7 and 20 can help to confirm intrahepatic cholangiocarcinoma and metastatic colon cancer. Our patient above was positive for both CK 7 and 20 suggesting that the tumor was likely hepatobiliary in origin [7].

Less than 30% of patients with hilar cholangiocarcinoma can undergo curative surgical resection successfully [6,8]. Other treatment options available in surgically unresectable patients include the placement of expandable metal wall stents to deliver palliative endoluminal brachytherapy and the placement of percutaneous external drains to decrease the high rate of cholangitis and occlusion [6]. There are also a plethora of chemotherapy and radiotherapy regimens available in the hopes of providing palliation and prolonging survival. The general prognosis of cholangiocarcinoma remains poor with studies showing a 24% five-year survival if the cancer is localized to the bile ducts and survival rates dropping to a mere 2% if cancer has distant metastasis [8,9].

The survival rate after diagnosis of metastatic disease in the setting of malignant effusions/ascites is a mere five months in patients with non-ovarian or breast malignancies [8,10]. Clinicians must recognize that malignancies can often masquerade as more indolent diseases; thorough evaluation is necessary to recognize these conditions. Ascitic fluid analysis is usually the first step in the workup of new-onset ascites to determine the etiology with cytological analysis reserved in cases where there is a high index of suspicion for malignancy. Clinicians should be aware that 8% of malignancy-related ascites can mimic SBP [5]. Therefore the appropriate treatment for these patients is not antibiotic therapy but rather chemotherapy or radiation therapy targeting underlying cancer. Biopsies are required to confirm the underlying primary cancer to help guide therapeutic decisions and determine prognosis.

## Conclusions

Our patient’s case presentation demonstrates the importance of utilizing the SAAG to guide the differential diagnosis and management of ascites. In the United States, 85% of patients will have ascites related to cirrhosis and portal hypertension, however, it is essential to devise a broad differential diagnosis in patients who develop new-onset ascites. A diagnostic paracentesis should be performed to evaluate the fluid cell count, albumin with ascitic cultures, and cytology. Calculating the SAAG score can help identify the presence of portal hypertension if greater than 1.1 g/dL. However, in patients with a SAAG less than 1.1 g/dL, additional diagnoses should be investigated including malignancy, pancreatitis, or nephrotic syndrome. Our patient in this case had a SAAG score of -0.4 g/dL and in the setting of a high index of suspicion of an underlying malignancy given her CT findings, history, physical and laboratory values, she underwent a liver biopsy and ascitic cytological analysis, which confirmed her diagnosis to be cholangiocarcinoma. The prognosis of malignancy-related ascites is generally very poor. The median survival after diagnosis can range from one to five months depending on the type of cancer and the extent of progression. Understanding the clinical significance of a SAAG less than 1.1 g/dL is vital in the workup of patients with new-onset ascites to help with a definite diagnosis and guide clinical management.
